# Supplementary material for: A CpG island-encoded mechanism protects genes from premature transcription termination
Source: Nat Commun. 2023 Feb 9;14:726. doi: 10.1038/s41467-023-36236-2 (PMC9911701; doi:10.1038/s41467-023-36236-2)
Supplement: Supplementary file 1 — Supplementary Information [file 41467_2023_36236_MOESM1_ESM.pdf]

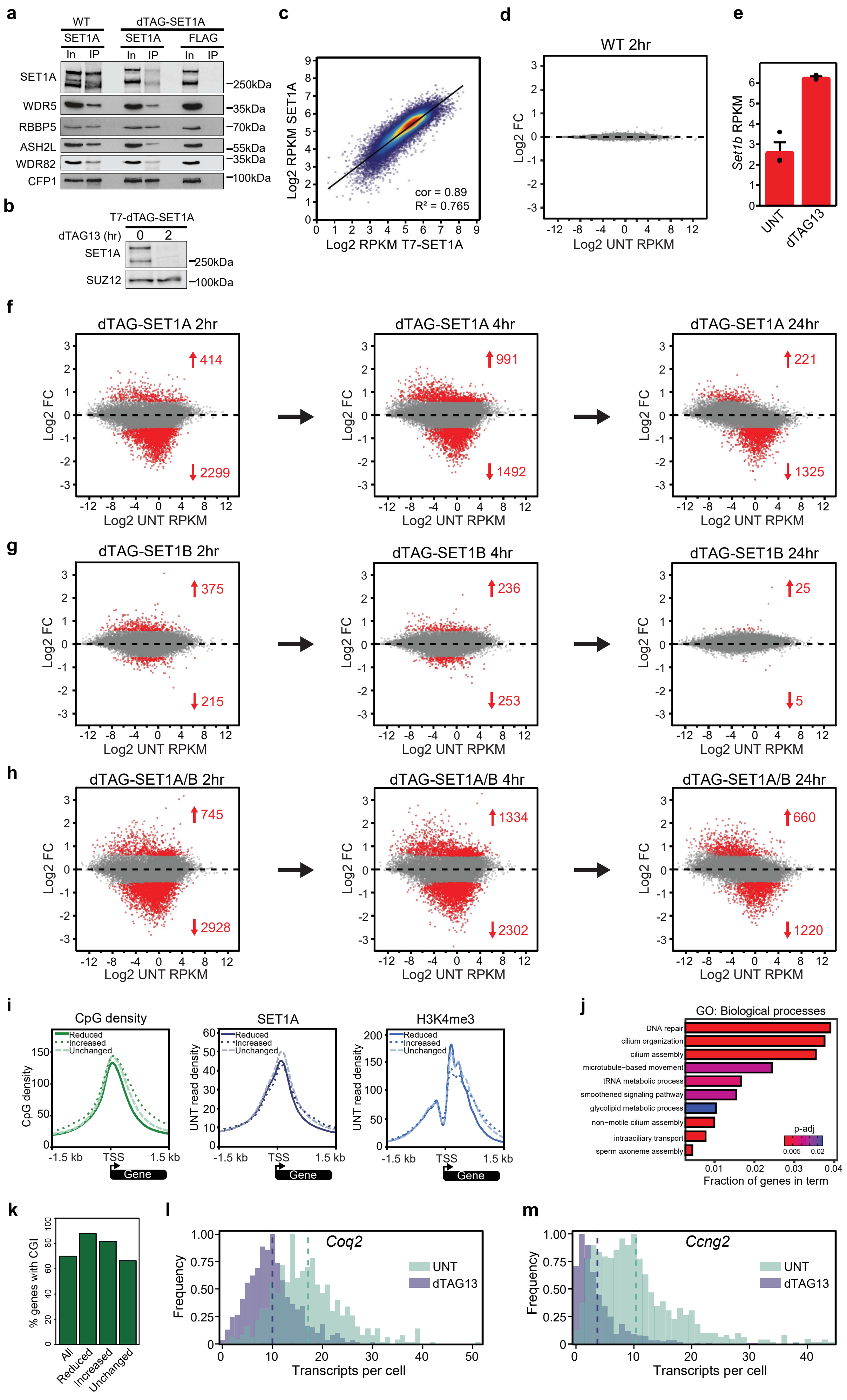

Supplementary Fig.1

Supplementary Fig.1

(a) Immunoprecipitation (IP) of SET1A from either wild type (WT) cells or dTAG-SET1A cells using a SET1A-specific antibody followed by western blot analysis of the nuclear extract input (In) and immunoprecipitated proteins, probed with the indicated antibodies. The FLAG antibody immunoprecipitation function as a control to indicate the specificity of the SET1A immunoprecipitations.
(b) A western blot showing T7-dTAG-SET1A levels before (0 hr) and following 2 hours of dTAG13 treatment. SUZ12 functions as a loading control.
(c) A scatter plot comparing the log2 RPKM of T7-dTAG-SET1A cChIP-seq signal and endogenous SET1A ChIP-seq [131] signal at CGIs that are within 1.5 kb of a TSS (n=15259). The spearman correlation (cor) and R^2 values are indicated.
(d) An MA-plot showing log2 fold changes (Log2 FC) in cRNA-seq signal in wild type ESCs following 2 hours of dTAG13 treatment (n=20633).
(e) A bar plot indicating the mean expression level (RPKM) of the *Set1b* gene before (UNT) and after 2 hours of dTAG13 treatment in the dTAG-SET1A cell line. Error bars represent SEM from three biological replicates.
(f) A series of MA-plots showing log2 fold changes (Log2 FC) in cRNA-seq signal in the dTAG-SET1A line following dTAG13 treatment for 2, 4, or 24 hours (n=20633), determined using DESeq2. Significant gene expression changes (p-adj<0.05 and >1.5-fold) are coloured red and the numbers of significantly changed genes are indicated.
(g) As per (f) but for the dTAG-SET1B line.
(h) As per (f) but for the dTAG-SET1A/B line.
(i) Metaplots comparing CpG density, SET1A levels, and H3K4me3 levels at the TSSs of CGI-associated genes that are reduced in expression after 2 hours of SET1A/B depletion (n=2571), increased in expression (n=609) or unchanged (n=11258).
(j) A gene ontology (GO) analysis of genes reduced in expression after 2 hours of SET1A/B depletion, determined using clusterProfiler [126].
(k) A bar plot showing the percentage of genes associated with CGIs comparing all genes (n=20633) to genes that are reduced in expression after 2 hours of SET1A/B depletion (n=2928), genes that are increased in expression (n=745) and those that are unchanged (n=16960).
(l-m) A histogram illustrating the number of transcripts per cell in the dTAG-SET1A/B line as measured by smRNA-FISH before (UNT, light green) and after 2 hours of dTAG13 treatment (light purple) for the (l) *Coq2* and (m) *Ccng2* genes. The dashed lines correspond to the mean of the distribution.

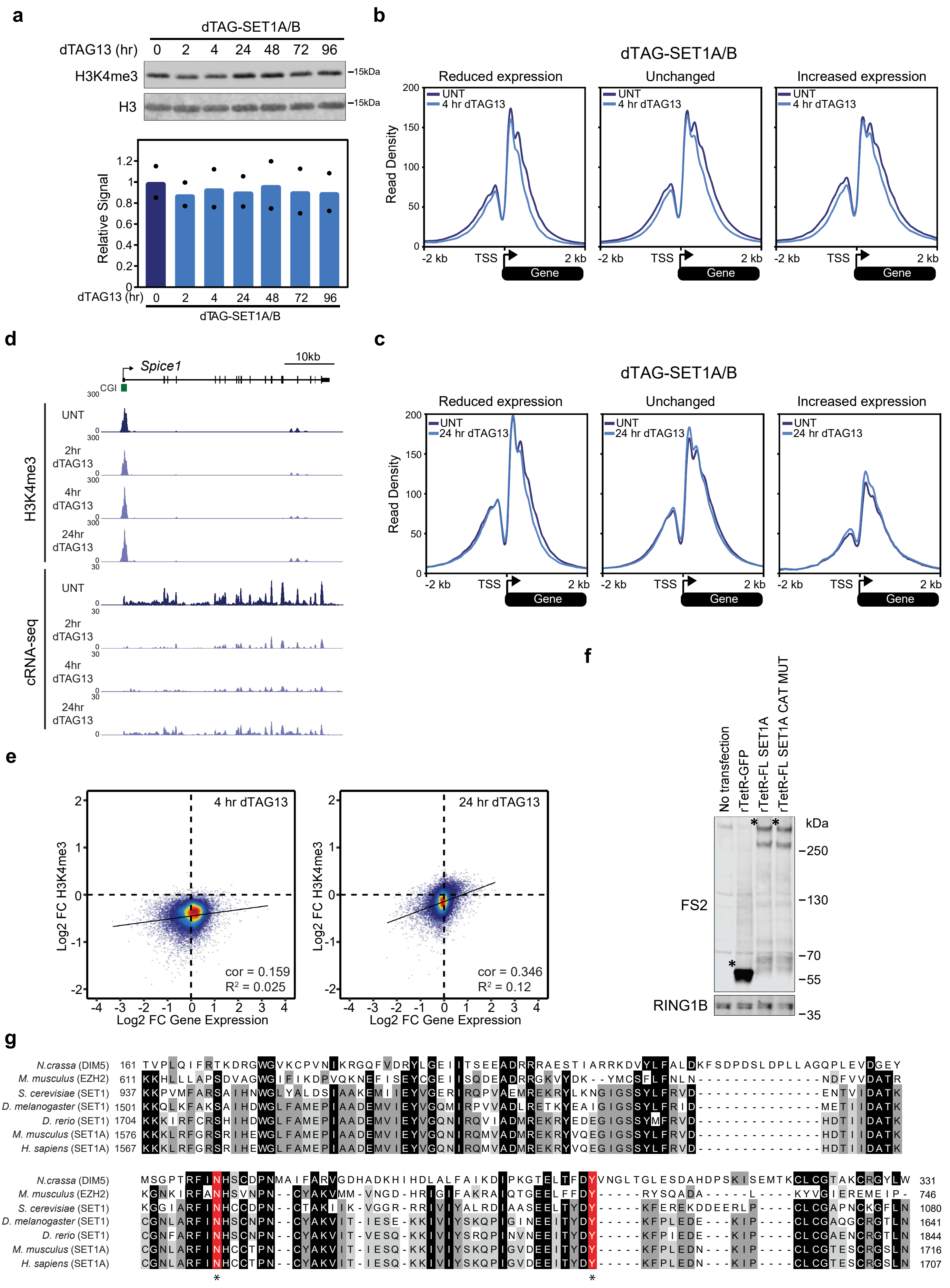

Supplementary Fig.2

Supplementary Fig.2

(a) Western blot analysis of H3K4me3 levels in the untreated dTAG-SET1A/B line and following 2, 4, 24, 48, 72, and 96 hours of dTAG13 treatment (top panels). H3 is included as a loading control. Mean H3K4me3 levels before and after dTAG13 treatment from 2 biological replicates is shown (right panel).
(b) Metaplot analysis of H3K4me3 cChIP-seq around the transcription start site (TSS) of genes that have reduced expression (left panel), unchanged expression (middle panel), or increased expression (right panel) in the dTAG-SET1A/B line before (UNT, dark blue lines) and after 4 hours of dTAG13 treatment (light blue lines). Only expressed genes are included (reduced expression, n=2050; unchanged, n=10081; increased expression, n=897).
(c) As in (b) but after 24 hour of dTAG13 treatment. Only expressed genes are included (reduced expression, n=1182; unchanged, n=11567; increased expression, n=279).
(d) A genomic snapshot comparing H3K4me3 cChIP-seq signal (top panels) and cRNA-seq (bottom panels) before and after 2, 4, and 24 hours of dTAG13 treatment in the dTAG-SET1A/B line at the *Spice1* gene.
(e) Scatter plots comparing the log2 fold change (Log2 FC) in H3K4me3 cChIP-seq signal and cRNA-seq signal (gene expression) in the dTAG-SET1A/B line after 4 hours (left panel) and 24 hours (right panel) of dTAG13 treatment. The pearson correlation (cor) and R^2 values are indicated. Only genes that have a peak of H3K4me3 in untreated cells are included (n=14065).
(f) A representative western blot (n = 2) against the FLAG-StrepII tag (FS2) showing expression of the rTetR fusion proteins used for reporter gene expression analysis in Fig.2. In each case the * indicates the position on the blot of the FS2-tagged rTetR fusion protein. RING1B is shown as a loading control. Molecular weight standards are shown in kilodaltons (kDa) on the right of the blot.
(g) A multiple sequence alignment of the SET domain of various histone methyltransferases from different species. Key catalytic residues that were mutated to inactivate SET1A methyltransferase activity are shown in red. Identical residues are highlighted in black and similar residues are highlighted in grey.

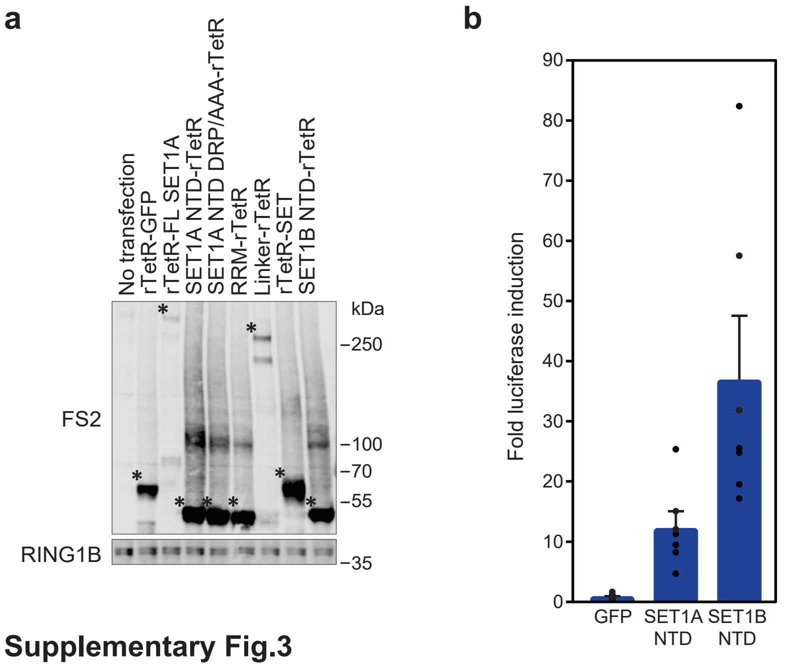

Supplementary Fig.3

Supplementary Fig.3

(a) A representative western blot (n = 2) against the FLAG-StrepII tag (FS2) showing expression of the various rTetR fusions used for reporter gene expression analysis in Fig.3 and Supplementary Fig.3. In each case the * indicates the position on the blot of the FS2-tagged rTetR fusion protein. RING1B is shown as a loading control. Molecular weight standards are shown in kilodaltons (kDa) on the right of the blot.

(b) A bar plot showing the mean fold induction of reporter gene expression after tethering of GFP, SET1A-NTD, and SET1B-NTD to the reporter gene. Error bars represent SEM from seven biological replicates.

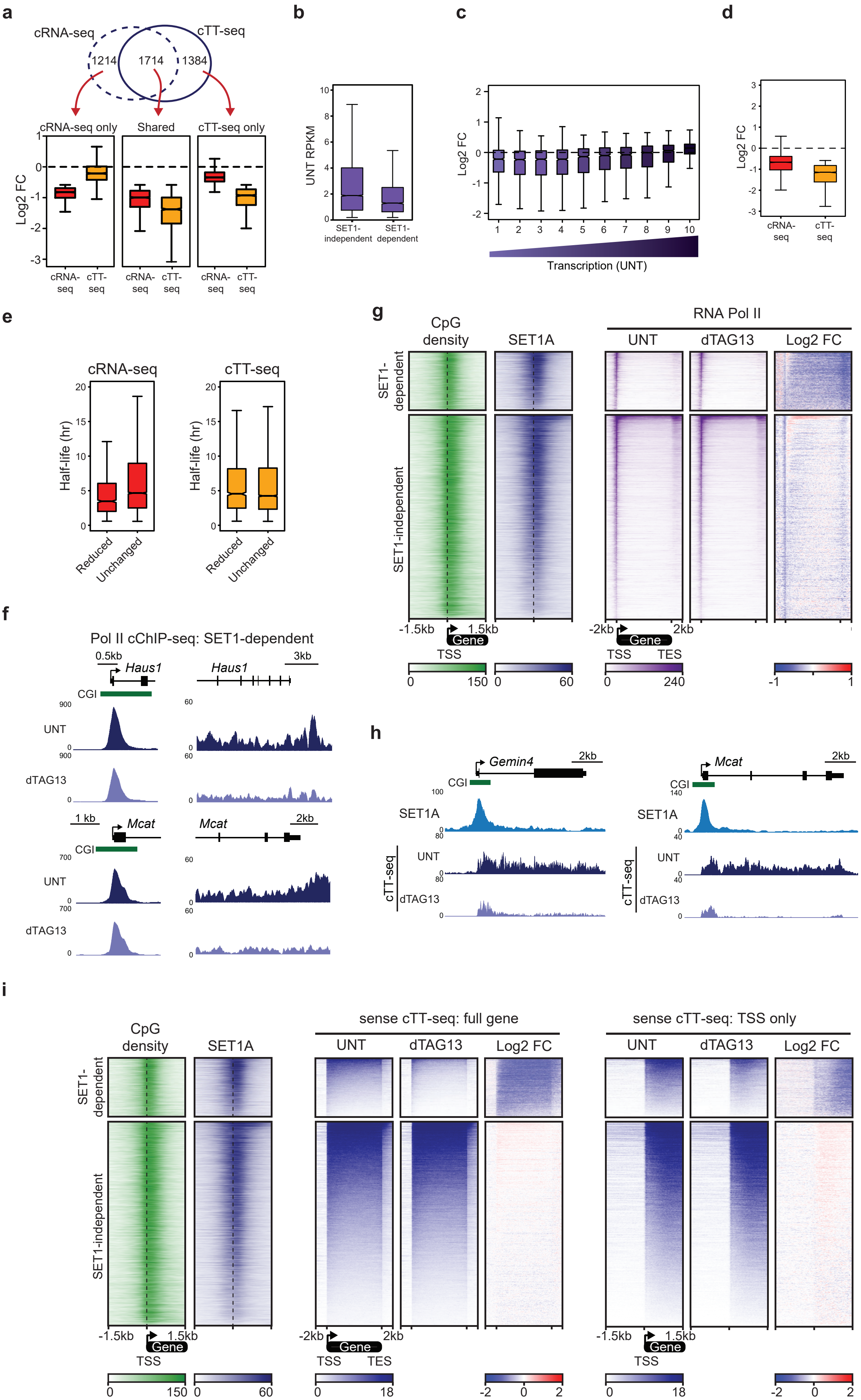

Supplementary Fig.4

Supplementary Fig.4

(a) A Venn diagram showing the overlap between genes significantly decreased following 2 hr SET1A/B depletion as measured by cRNA-seq or cTT-seq (top panel). Box plots of log2 fold change (Log2 FC) in gene expression, as measured using either cRNA-seq or cTT-seq, of all significantly decreased genes after 2 hours of SET1A/B depletion (bottom panel). Genes are split into three groups, depending on whether they were significantly decreased in cRNA-seq only, cTT-seq only or in both cRNA-seq and cTT-seq (Shared). The number of genes in each subset are as per the Venn diagram. The boxes show interquartile range, centre line represents median, whiskers extend by 1.5x IQR or the most extreme point (whichever is closer to the median), while notches extend by 1.58x IQR/sqrt(n), giving a roughly 95% confidence interval for comparing medians.
(b) A box plot showing the level of transcription in untreated cells (UNT RPKM) for actively transcribed SET1-dependent (n=2633) and SET1-independent genes (n=9151). Boxes are defined as in a.
(c) A box pot showing log2 fold change (Log2 FC) in transcription in the dTAG-SET1A/B line after 2 hours dTAG13 treatment with transcribed genes (n=11823) separated into deciles based on their transcription level in untreated cells. Boxes are defined as in a.
(d) A box plot showing log2 fold change (Log2 FC) in gene expression, as measured using either cRNA-seq or cTT-seq, at all actively transcribed genes with significantly reduced transcription after 2 hours of SET1A/B depletion (n=2633). Boxes are defined as in a.
(e) Left panel: A box plot showing the transcript half-life for genes with reduced expression in cRNA-seq when SET1A/B are depleted (n=1238) and those that are unchanged (n=6256). The right panel is a box plot showing the transcript half-life for genes with reduced transcription in cTT-seq when SET1A/B are depleted (n=1502) and those that are unchanged (n=6265). Only genes with an annotated half-life from [132] are included (n=7788). Boxes are defined as in a.
(f) Genomic snapshots of RNA Pol II occupancy (Pol II cChIP-seq) at two SET1-dependent genes (*Haus1* and *Mcat*) in dTAG-SET1A/B cells that are untreated (UNT, dark purple) or treated with dTAG13 for 2 hours (light purple). The left hand panels correspond to gene promoter occupancy and the right hand panels to gene body occupancy. CGIs are shown in green.
(g) Heatmaps illustrating CpG density and SET1A occupancy at transcribed SET1-independent (n=9151) and SET1-dependent (n=2633) gene TSSs ranked by transcription level in untreated cells (left panels). The right panel contains heatmaps illustrating RNA Pol II occupancy over the same genes before and after 2 hours of dTAG13 treatment, with the log2 fold change (Log2 FC) in RNA Pol II also shown.
(h) Genomic snapshots of cTT-seq signal in the dTAG-SET1A/B line at two SET1-dependent genes (*Gemin4* and *Mcat*) in untreated cells (UNT, dark purple) or cells treated with dTAG13 for 2 hours (light purple). The location of CGIs is shown in green and SET1A cChIP-seq signal in light blue.
(i) Heatmaps illustrating CpG density and SET1A occupancy at transcribed SET1-independent (n=9151) and SET1-dependent (n=2633) gene TSSs ranked by transcription level in untreated cells (left panels). The right panels contain heatmaps illustrating sense cTT-seq signal over the same genes before (UNT) and after 2 hours of dTAG13 treatment. Heatmaps show either the full gene body (middle panels) or just the TSS region (right panels). The log2 fold change (Log2 FC) in cTT-seq signal is also shown.

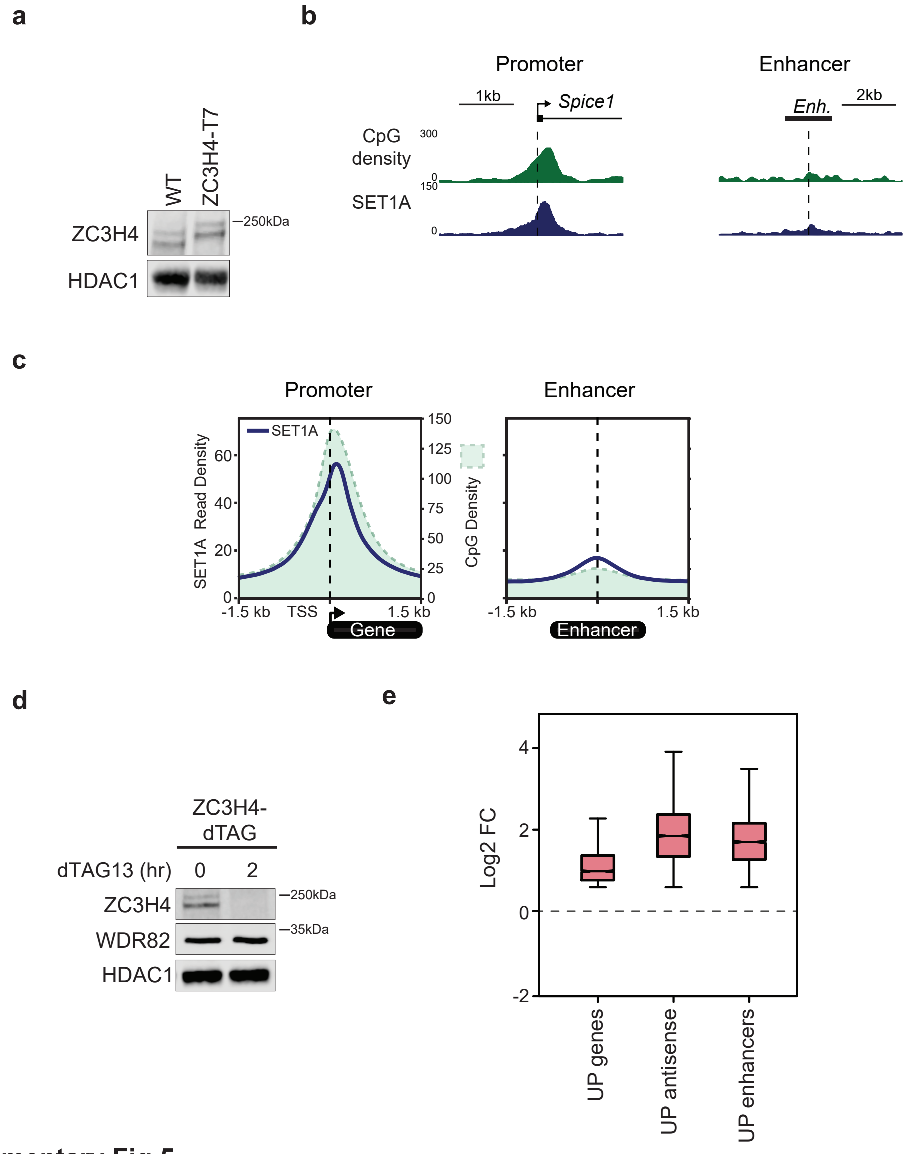

Supplementary Fig.5

Supplementary Fig.5

(a) A representative western blot (n = 3) for ZC3H4 in WT and ZC3H4-T7 cell lines. HDAC1 functions as a loading control.
(b) Genomic snapshots illustrating CpG density and SET1A cChIP-seq signal at the promoter of the *Spice1* gene (left panel) and an intergenic enhancer region (chr16:30,818,164-30,824,484) (right panel).
(c) Metaplot analysis of CpG density (green shaded area) and SET1A cChIP-seq (blue line) at transcribed gene promoters (n=11823, left panel) and intergenic enhancers (n=4156, right panel). The read density of SET1A is shown on the left axis and CpG density is shown on the right axis.
(d) A western blot for ZC3H4 and WDR82 in the ZC3H4-dTAG line, both untreated (0 hr) and treated with dTAG13 for 2 hr. HDAC1 functions as a loading control.
(e) A box pot showing the log2 fold change (Log2 FC) in transcription in the ZC3H4-dTAG line after 2 hours dTAG13 treatment at genes with increased transcription (UP genes, n=2599), TSS antisense regions with increased transcription (UP antisense, n=8455), and enhancers with increased transcription (UP enhancers, n=2993). The boxes show interquartile range, centre line represents median, whiskers extend by 1.5x IQR or the most extreme point (whichever is closer to the median), while notches extend by 1.58x IQR/sqrt(n), giving a roughly 95% confidence interval for comparing medians.

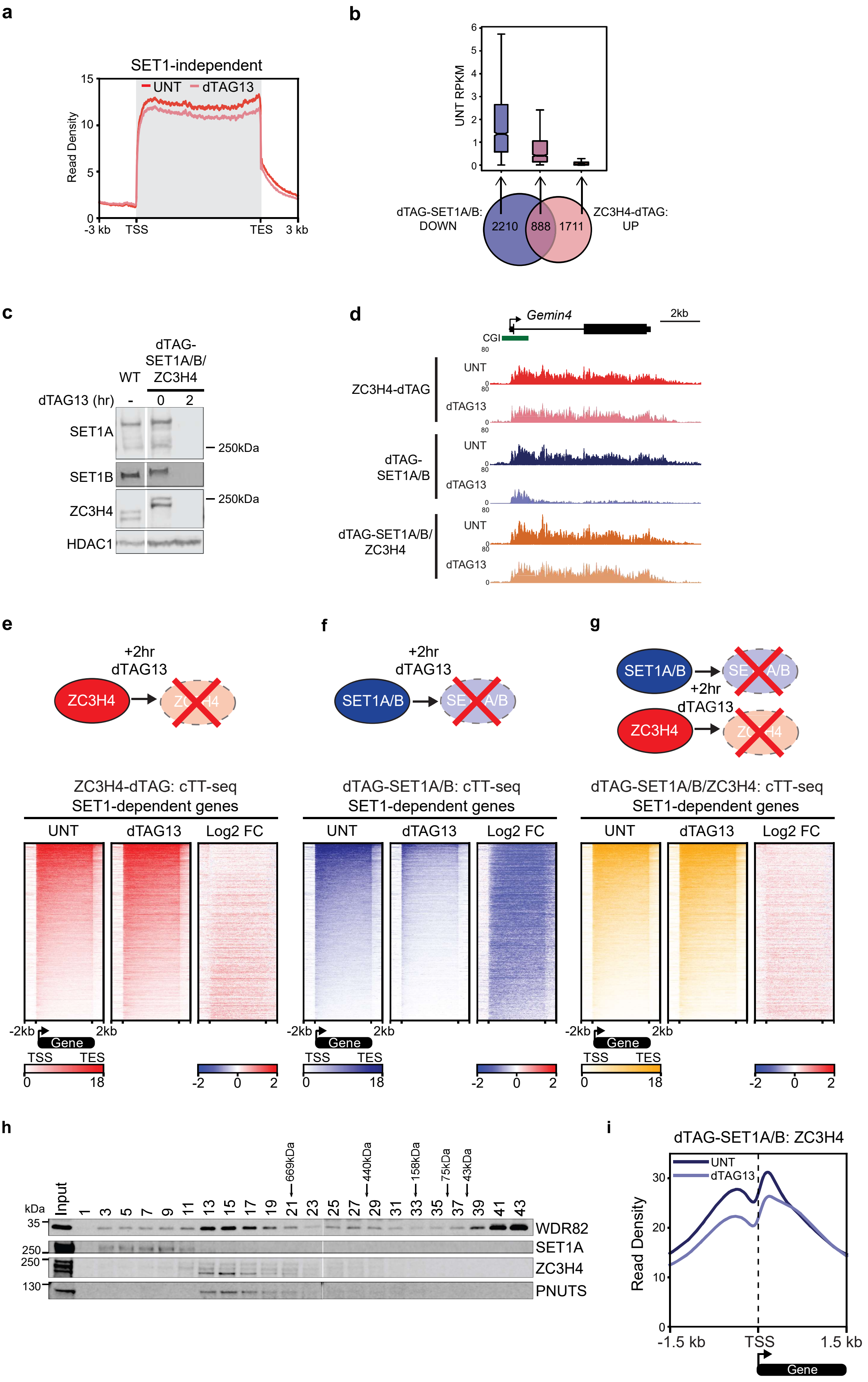

Supplementary Fig.6

Supplementary Fig.6

(a) Metaplot analysis of transcription (cTT-seq) in the ZC3H4-dTAG line that is either untreated (UNT) or treated with dTAG13 for 2 hours at transcribed SET1-independent genes (n=9151).
(b) A Venn diagram showing the overlap between genes with significantly reduced transcription following 2 hr SET1A/B depletion and genes with significantly increased transcription following 2 hr ZC3H4 depletion (bottom panel). A box plot showing the level of transcription in untreated cells (UNT RPKM) of the genes with significantly reduced transcription following SET1A/B depletion or significantly increased transcription following ZC3H4 depletion (top panel). The number of genes in each subset are as per the Venn diagram. The boxes show interquartile range, centre line represents median, whiskers extend by 1.5x IQR or the most extreme point (whichever is closer to the median), while notches extend by 1.58x IQR/sqrt(n), giving a roughly 95% confidence interval for comparing medians.
(c) A representative western blot (n = 2) for SET1A, SET1B and ZC3H4 in WT cells and the dTAG-SET1A/B/ZC3H4 line before (0 hr) and after 2 hr treatment with dTAG13. HDAC1 functions as a loading control.
(d) A genomic snapshot of cTT-seq signal in the ZC3H4-dTAG, dTAG-SET1A/B and dTAG-SET1A/B/ZC3H4 lines at a SET1-dependent gene (*Gemin4*) in untreated cells (UNT) or cells treated with dTAG13 for 2 hours.
(e) A schematic illustrating the ZC3H4-dTAG line (top panel) and a heatmap showing cTT-seq signal at transcribed SET1-dependent genes (n=2633) in untreated cells (UNT) or cells treated with dTAG13 for 2 hours (bottom panel). The log2 fold change (Log2 FC) in cTT-seq signal is also shown. Heatmaps are sorted by transcription in untreated cells.
(f) As per (e) but for the dTAG-SET1A/B line.
(g) As per (e) but for the dTAG-SET1A/B/ZC3H4 line.
(h) Size exclusion chromatography analysis of WT ESC nuclear extract probed by western blot with the indicated antibodies. Elution positions of known MW standards are marked.
(i) Metaplot analysis of ZC3H4 ChIP-seq signal at all TSSs (n=20633) in the dTAG-SET1A/B line that is either untreated (UNT) or treated with dTAG13 for 2 hours.

Set1a	TAAATGAGCAAAGATGGACC
Set1b	TTCAGGTTGGGTTAACGGCA
Zc3h4	CCTTCTGCCAGTAGTGTCAC

Supplementary Table 1

sgRNA sequences used in this study.

Antibody	Source	Catalogue Number	Application	Western dilution	ChIP/IP amount
anti-SET1A	Bethyl Laboratories	A300-289A	Western blot	1/500	N/A
anti-SET1B D1U5D	Cell Signaling	44922	Western blot	1/500	N/A
anti-SUZ12 D39F6	Cell Signaling	3737	Western blot	1/1000	N/A
anti-WDR5 D9E1I	Cell Signaling	13105	Western blot	1/1000	N/A
anti-RBBP5 D3I6P	Cell Signaling	13171	Western blot	1/1000	N/A
anti-ASH2L D93F6	Cell Signaling	5019	Western blot	1/1000	N/A
anti-WDR82 D2I3B	Cell Signaling	99715	Western blot	1/1000	N/A
anti-CFP1	Klose Lab (Brown et al. 2017)	N/A	Western blot	1/2000	N/A
anti-H3K4me3	Klose Lab	N/A	Western blot and ChIP	1/2000	1.5 µl
anti-H3 96C10	Cell Signaling	3638	Western blot	1/2000	N/A
anti-FS2	Klose Lab	N/A	Western blot	1/500	N/A
anti-RING1B	Atsuta et al. 2001	N/A	Western blot	1/1000	N/A
anti-FLAG	Sigma	A8592	Western blot	1/500	N/A
anti-HDAC1	Abcam	ab109411	Western blot	1/3000	N/A
anti-ZC3H4	Atlas Antibodies	HPA040934	Western blot	1/500	N/A
anti-PNUTS	Cell Signaling	14171	Western blot	1/1000	N/A
anti-SET1A	Klose Lab	N/A	IP	N/A	20 µl
anti-FLAG	Sigma	F1804	IP	N/A	5 µl
anti-T7 D9E1X	Cell Signaling	13246	ChIP	N/A	10 µl
anti-Rbp1-NTD D8L4Y	Cell Signaling	14958	ChIP	N/A	15 µl
IRDye 800CW Goat anti-Mouse IgG	LI-COR	926-32210	Western blot	1/15000	N/A
IRDye 800CW Goat anti-Rabbit IgG	LI-COR	926-32211	Western blot	1/15000	N/A
IRDye 680RD Goat anti-Mouse IgG	LI-COR	926-68070	Western blot	1/15000	N/A
IRDye 680RD Goat anti-Rabbit IgG	LI-COR	926-68071	Western blot	1/15000	N/A
Anti-rabbit secondary (HRP)	VWR	NA934	Western blot	1/2500	N/A
Anti-mouse secondary (HRP)	VWR	NA931	Western blot	1/2500	N/A
VeriBlot for IP Detection Reagent (HRP)	Abcam	ab131366	Western blot	1/500	N/A

Supplementary Table 2

A list of antibodies used in this study.
