# Supplementary material for: A CpG island-encoded mechanism protects genes from premature transcription termination
Source: Nat Commun. 2023 Feb 9;14:726. doi: 10.1038/s41467-023-36236-2 (PMC9911701; doi:10.1038/s41467-023-36236-2)
Supplement: Supplementary file 3 — Description of additional Supplementary File [file 41467_2023_36236_MOESM3_ESM.pdf]

Descriptions of additional supplementary files

Supplementary Data 1

Total numbers of uniquely mapped, non-duplicate reads for mouse and spike-in genomes from all cRNA-seq, cTT-seq and cChIP-seq experiments performed in this study.
