# Supplementary material for: A CpG island-encoded mechanism protects genes from premature transcription termination
Source: Nat Commun. 2023 Feb 9;14:726. doi: 10.1038/s41467-023-36236-2 (PMC9911701; doi:10.1038/s41467-023-36236-2)
Supplement: Supplementary file 5 — Reporting Summary [file 41467_2023_36236_MOESM5_ESM.pdf]

nature portfolio

Corresponding author(s): Robert J. Klose

Last updated by author(s): 15/12/22

Reporting Summary

Nature Portfolio wishes to improve the reproducibility of the work that we publish. This form provides structure for consistency and transparency in reporting. For further information on Nature Portfolio policies, see our Editorial Policies and the Editorial Policy Checklist.

Statistics

For all statistical analyses, confirm that the following items are present in the figure legend, table legend, main text, or Methods section.

n/a	Confirmed	
☐	☒	The exact sample size (*n*) for each experimental group/condition, given as a discrete number and unit of measurement
☐	☒	A statement on whether measurements were taken from distinct samples or whether the same sample was measured repeatedly
☐	☒	The statistical test(s) used AND whether they are one- or two-sided<br>*Only common tests should be described solely by name; describe more complex techniques in the Methods section.*
☒	☐	A description of all covariates tested
☒	☐	A description of any assumptions or corrections, such as tests of normality and adjustment for multiple comparisons
☐	☒	A full description of the statistical parameters including central tendency (e.g. means) or other basic estimates (e.g. regression coefficient) AND variation (e.g. standard deviation) or associated estimates of uncertainty (e.g. confidence intervals)
☐	☒	For null hypothesis testing, the test statistic (e.g. *F*, *t*, *r*) with confidence intervals, effect sizes, degrees of freedom and *P* value noted<br>*Give P values as exact values whenever suitable.*
☒	☐	For Bayesian analysis, information on the choice of priors and Markov chain Monte Carlo settings
☒	☐	For hierarchical and complex designs, identification of the appropriate level for tests and full reporting of outcomes
☐	☒	Estimates of effect sizes (e.g. Cohen's *d*, Pearson's *r*), indicating how they were calculated

Our web collection on statistics for biologists contains articles on many of the points above.

Software and code

Policy information about availability of computer code

Data collection	Image Studio v5.2
Data analysis	R version 4.0.2 (R Core Team, 2020), Bowtie 2 v2.3.4.1 (Langmead and Salzberg, 2012), STAR v2.5.4 (Dobin et al., 2013), MACS2 v2.1.1 (Zhang et al., 2008), Sambamba v0.6.7 (Tarasov et al., 2015), SAMtools v1.7 (Li et al., 2009), deepTools v3.1.1 (Ramirez et al., 2016), BEDtools v2.17.0 (Quinlan and Hall, 2010), DESeq2 1.22.2 (Love et al., 2014), clusterProfiler (Yu G et al. 2012), ImageJ 1.52p through Fiji (Schindelin et al., 2012), MUSCLE v5 (Edgar et al. 2004), Jalview 2.11.2.5 (Waterhouse et al. 2009), https://github.com/nFursova/Calibrated_ChIPseq_RNAseq, ThunderFISH (https://github.com/aleks-szczure/ThunderFISH)

For manuscripts utilizing custom algorithms or software that are central to the research but not yet described in published literature, software must be made available to editors and reviewers. We strongly encourage code deposition in a community repository (e.g. GitHub). See the Nature Portfolio guidelines for submitting code & software for further information.

Data

Policy information about availability of data

All manuscripts must include a data availability statement. This statement should provide the following information, where applicable:

- Accession codes, unique identifiers, or web links for publicly available datasets
- A description of any restrictions on data availability
- For clinical datasets or third party data, please ensure that the statement adheres to our policy

High-throughput sequencing datasets generated for this study are available in the GEO database under the accession number (GSE199805). Published data used in

this study include BioCAP-seq data (GSE43512), enhancer annotations (GSE161996), CpG density tracks (King et al. 2018) SET1A ChIP-seq (GSE98140) and mRNA half-life data (GSE86336). For cnRNA-seq processing we used mm10 (GenBank: BK000964.3, https://www.ncbi.nlm.nih.gov/nuccore/bk000964) and dm6 (GenBank: M21017.1, https://www.ncbi.nlm.nih.gov/nuccore/M21017.1) rDNA genomic datasets.

Human research participants

Policy information about studies involving human research participants and Sex and Gender in Research.

Reporting on sex and gender	Not applicable.
Population characteristics	Not applicable.
Recruitment	Not applicable.
Ethics oversight	Not applicable.

Note that full information on the approval of the study protocol must also be provided in the manuscript.

Field-specific reporting

Please select the one below that is the best fit for your research. If you are not sure, read the appropriate sections before making your selection.

[x] Life sciences [ ] Behavioural & social sciences [ ] Ecological, evolutionary & environmental sciences

For a reference copy of the document with all sections, see nature.com/documents/nr-reporting-summary-flat.pdf

Life sciences study design

All studies must disclose on these points even when the disclosure is negative.

Sample size	Sample sizes were determined based on previous studies performed in the lab using similar techniques to enable reasonable statistical analysis (Rose et al. 2016; Fursova et al. 2019; Turberfield et al. 2019; Blackledge et al. 2020, Dobrinic et al. 2021).
Data exclusions	No data were excluded.
Replication	Reported experimental findings were reproducible in multiple independent biological replicates. All RNA-seq, TT-seq and ChIP-seq experiments were all performed in at least biological triplicate. The numbers of biological replicates for each experiment are given in the Methods section and/or in the figure legends.
Randomization	Randomization was not relevant for this study as it includes only molecular assays performed in cell lines of known genotype.
Blinding	Blinding was not relevant for this study as there were no prior assumptions about experimental outcomes. All data was collected and processed uniformly regardless of treatment groups.

Reporting for specific materials, systems and methods

We require information from authors about some types of materials, experimental systems and methods used in many studies. Here, indicate whether each material, system or method listed is relevant to your study. If you are not sure if a list item applies to your research, read the appropriate section before selecting a response.

Materials & experimental systems

n/a	Involved in the study	
[ ]	[x]	Antibodies
[ ]	[x]	Eukaryotic cell lines
[x]	[ ]	Palaeontology and archaeology
[x]	[ ]	Animals and other organisms
[x]	[ ]	Clinical data
[x]	[ ]	Dual use research of concern

Methods

n/a	Involved in the study	
[ ]	[x]	ChIP-seq
[x]	[ ]	Flow cytometry
[x]	[ ]	MRI-based neuroimaging

Antibodies

Antibodies used	Antibody Source Catalogue Number Application Western dilution ChIP/IP amount

c Western blot 1/500 N/A
anti-SET1B D1U5D Cell Signaling 44922 Western blot 1/500 N/A
anti-SUZ12 D39F6 Cell Signaling 3737 Western blot 1/1000 N/A
anti-WDR5 D9E1I Cell Signaling 13105 Western blot 1/1000 N/A
anti-RBBP5 D3I6P Cell Signaling 13171 Western blot 1/1000 N/A
anti-ASH2L D93F6 Cell Signaling 5019 Western blot 1/1000 N/A
anti-WDR82 D2I3B Cell Signaling 99715 Western blot 1/1000 N/A
anti-CFP1 Klose Lab (Brown et al. 2017) N/A Western blot 1/2000 N/A
anti-H3K4me3 Klose Lab N/A Western blot and ChIP 1/2000 1.5 µl
anti-H3 96C10 Cell Signaling 3638 Western blot 1/2000 N/A
anti-FS2 Klose Lab N/A Western blot 1/500 N/A
anti-RING1B Atsuta et al. 2001 N/A Western blot 1/1000 N/A
anti-FLAG Sigma A8592 Western blot 1/500 N/A
anti-HDAC1 Abcam ab109411 Western blot 1/3000 N/A
anti-ZC3H4 Atlas Antibodies HPA040934 Western blot 1/500 N/A
anti-PNUTS Cell Signalling 14171 Western blot 1/1000 N/A
anti-SET1A Klose Lab N/A IP N/A 20 µl
anti-FLAG Sigma F1804 IP N/A 5 µl
anti-T7 D9E1X Cell Signaling 13246 ChIP N/A 10 µl
anti-Rbp1-NTD D8L4Y Cell Signaling 14958 ChIP N/A 15 µl
IRDye 800CW Goat anti-Mouse IgG LI-COR 926-32210 Western blot 1/15000 N/A
IRDye 800CW Goat anti-Rabbit IgG LI-COR 926-32211 Western blot 1/15000 N/A
IRDye 680RD Goat anti-Mouse IgG LI-COR 926-68070 Western blot 1/15000 N/A
IRDye 680RD Goat anti-Rabbit IgG LI-COR 926-68071 Western blot 1/15000 N/A
Anti-rabbit secondary (HRP) VWR NA934 Western blot 1/2500 N/A
Anti-mouse secondary (HRP) VWR NA931 Western blot 1/2500 N/A
VeriBlot for IP Detection Reagent (HRP) Abcam ab131366 Western blot 1/500 N/A

Validation

anti-SET1A (Bethyl) - Manufacturer-validated in human cells by western blot and ChIP, validated in the Klose lab in a conditional knock-out mouse ESC line by western blot (unpublished) and in this manuscript in a degron mouse ESC line by western blot. 53 citations - https://www.citeab.com/antibodies/654777-a300-289a-rabbit-anti-hset1-antibody-affinity-purifi
anti-SET1B - Manufacturer-validated by western blot in human and mouse cells, validated in the Klose lab using a knockout mouse ESC cell line by western blot (unpublished) and validated in this manuscript in a degron mouse ESC line by western blot. 1 citation - https://www.citeab.com/antibodies/4671100-44922-set1b-d1u5d-rabbit-mab?des=cd31b6a95c317d8f
anti-SUZ12 - Manufacturer-validated in mouse and human cell lines by western blot, validated in the Klose lab by ChIP and in a degron mouse ESC cell line by western blot (Dobrinic et al. 2020).
anti-WDR5 - Manufacturer-validated by ChIP and in various human and mouse cell lines by western blot. Validated in this manuscript in mouse ESCs by SET1A co-IP. 32 citations - https://www.citeab.com/antibodies/2043094-13105-wdr5-d9e1i-rabbit-mab?des=0572588a1079e430
anti-RBBP5 - Manufacturer-validated in various human and mouse cell lines by western blot. Validated in this manuscript in mouse ESCs by SET1A co-IP. 12 citations - https://www.citeab.com/antibodies/2043107-13171-rbbp5-d3i6p-rabbit-mab?des=1e6f7b72d242f623
anti-ASH2L - Manufacturer-validated in various human and mouse cell lines by western blot. Validated in this manuscript in mouse ESCs by SET1A co-IP. 15 citations - https://www.citeab.com/antibodies/125001-5019-ash2l-d93f6-xp-rabbit-mab?des=80a9cfcc9b539186
anti-WDR82 - Manufacturer-validated in various human and mouse cell lines by western blot. Validated in this manuscript in mouse ESCs by SET1A co-IP. Validated in the Klose Lab using a degron mouse ESC cell line (unpublished). 9 citations - https://www.citeab.com/antibodies/3394276-99715-wdr82-d2i3b-rabbit-mab?des=2a91774e08c198ba
anti-CFP1 - Validated in the Klose Lab in a mouse ESC conditional knockout cell line (Brown et al. 2017).
anti-H3K4me3 - Validated in the Klose Lab using differentially-methylated nucleosomes in ChIP (Epicypher, Shah et al. 2018)
anti-H3 - Manufacturer-validated in various human and mouse cell lines by western blot. 182 citations - https://www.citeab.com/antibodies/123216-3638-histone-h3-96c10-mouse-mab?des=06be782c4c65f03e
anti-FS2 - Validated in the Klose Lab in mouse ESCs by western blot against FS2-tagged proteins (Blackledge et al. 2014)
anti-RING1B - Validated in Atsuta et al., 2001
anti-FLAG (Sigma A8592) - Validated in the Klose Lab in mouse ESCs by western blot against FLAG-tagged proteins. 1771 citations - https://www.citeab.com/antibodies/1038440-a8592-monoclonal-anti-flag-r-m2-peroxidase-hrp-an
anti-HDAC1 - Manufacturer-validated in various human and mouse cell lines by western blot and immunofluorescence. 39 citations - https://www.citeab.com/antibodies/762876-ab109411-anti-hdac1-antibody-epr460-2?des=a30c4f49a72104c7
anti-ZC3H4 Manufacturer-validated using siRNA depletion in human cells by western blot, validated in the Klose lab in this manuscript in both a degron and epitope-tagged mouse ESC line by western blot. 1 citation - https://www.citeab.com/antibodies/148045-hpa040934-anti-zc3h4-zinc-finger-ccch-type-containin?des=e9f2d8481c1692f1
anti-SET1A (Klose Lab) - Validated for western and IP in the Klose Lab in this manuscript using a mouse ESC degron cell line and SET1A conditional knockout mouse ESC line (unpublished).
anti-T7 - Manufacturer-validated against T7-tagged proteins. Validated in this manuscript by western blot and ChIP in a T7-SET1A degron cell line. Validated for ChIP-seq in the Klose Lab (Brown et al. 2017).
anti-Rbp1-NTD - Manufacturer-validated in various cell lines by western blot. Validated in the Klose Lab for ChIP (Dobrinic et al. 2020, Turberfield et al. 2019)
anti-PNUTS - Manufacturer-validated in various human cell lines by western blot. Validated in the Klose Lab by western blotting in PNUTS-T7 mouse ESCs (unpublished). 1 citation: https://www.citeab.com/antibodies/2397784-14171-pnuts-antibody?

des=1664d4bef2506019
anti-FLAG (Sigma F1804) - Validated in the Klose Lab in mouse ESCs for IP of FLAG-tagged proteins. 7638 citations - https://www.citeab.com/antibodies/2304935-f1804-monoclonal-anti-flag-r-m2-antibody-produced-i?des=ff55dc14225e3731

Eukaryotic cell lines

Policy information about cell lines and Sex and Gender in Research

Cell line source(s)	All ESC cell lines used in this study were generated in the Klose lab:<br>dTAG-SET1A mouse embryonic stem cell line generated in this study.<br>dTAG-SET1B mouse embryonic stem cell line generated in this study.<br>dTAG-SET1A/B mouse embryonic stem cell line generated in this study.<br>ZC3H4-dTAG mouse embryonic stem cell line generated in this study.<br>dTAG-SET1A/B/ZC3H4 mouse embryonic stem cell line generated in this study.<br>T7-dTAG-SET1A mouse embryonic stem cell line generated in this study.<br>dTAG-SET1A/B; ZC3H4-T7 mouse embryonic stem cell line generated in this study.<br>TOT2N mouse embryonic stem cell line (with TetO integration) (Blackledge et al., 2014). Modified with luciferase reporter gene in this study.<br>Human HEK293T or drosophila SG4 cells (sourced from ATCC) were used as material for calibration but not as an experimental system. HEK293T T7-SCC1 cell line used for calibration was provided by the Kim Nasmyth lab.
Authentication	All cell lines generated in this study were validated by PCR, sequencing and Western blot. All cell lines generated for previous studies (Blackledge et al, 2014) were validated in their respective publication.
Mycoplasma contamination	All cell lines were regularly tested for mycoplasma contamination and confirmed to be negative.
Commonly misidentified lines<br>(See ICLAC register)	No commonly misidentified cell lines were used.

ChIP-seq

Data deposition

☒ Confirm that both raw and final processed data have been deposited in a public database such as GEO.

☒ Confirm that you have deposited or provided access to graph files (e.g. BED files) for the called peaks.

Data access links<br>*May remain private before publication.*	GSE199805
Files in database submission	T7SET1AChIP_Rep10hr_R1.fastq.gz<br>T7SET1AChIP_Rep10hr_R2.fastq.gz<br>T7SET1AChIP_Rep12hr_R1.fastq.gz<br>T7SET1AChIP_Rep12hr_R2.fastq.gz<br>T7SET1AChIP_Rep20hr_R1.fastq.gz<br>T7SET1AChIP_Rep20hr_R2.fastq.gz<br>T7SET1AChIP_Rep22hr_R1.fastq.gz<br>T7SET1AChIP_Rep22hr_R2.fastq.gz<br>T7SET1AChIP_Rep30hr_R1.fastq.gz<br>T7SET1AChIP_Rep30hr_R2.fastq.gz<br>T7SET1AChIP_Rep32hr_R1.fastq.gz<br>T7SET1AChIP_Rep32hr_R2.fastq.gz<br>Input_T7SET1AChIP_Rep10hr_R1.fastq.gz<br>Input_T7SET1AChIP_Rep10hr_R2.fastq.gz<br>Input_T7SET1AChIP_Rep12hr_R1.fastq.gz<br>Input_T7SET1AChIP_Rep12hr_R2.fastq.gz<br>Input_T7SET1AChIP_Rep20hr_R1.fastq.gz<br>Input_T7SET1AChIP_Rep20hr_R2.fastq.gz<br>Input_T7SET1AChIP_Rep22hr_R1.fastq.gz<br>Input_T7SET1AChIP_Rep22hr_R2.fastq.gz<br>Input_T7SET1AChIP_Rep30hr_R1.fastq.gz<br>Input_T7SET1AChIP_Rep30hr_R2.fastq.gz<br>Input_T7SET1AChIP_Rep32hr_R1.fastq.gz<br>Input_T7SET1AChIP_Rep32hr_R2.fastq.gz<br>T7SET1AChIP_0hrMERGE.bw<br>T7SET1AChIP_2hrMERGE.bw<br>Z4ChIP_Rep10hr_R1.fastq.gz<br>Z4ChIP_Rep10hr_R2.fastq.gz<br>Z4ChIP_Rep12hr_R1.fastq.gz

Z4ChIP_Rep12hr_R2.fastq.gz
Z4ChIP_Rep20hr_R1.fastq.gz
Z4ChIP_Rep20hr_R2.fastq.gz
Z4ChIP_Rep22hr_R1.fastq.gz
Z4ChIP_Rep22hr_R2.fastq.gz
Z4ChIP_Rep30hr_R1.fastq.gz
Z4ChIP_Rep30hr_R2.fastq.gz
Z4ChIP_Rep32hr_R1.fastq.gz
Z4ChIP_Rep32hr_R2.fastq.gz
INPUT_Z4ChIP_Rep10hr_R1.fastq.gz
INPUT_Z4ChIP_Rep10hr_R2.fastq.gz
INPUT_Z4ChIP_Rep12hr_R1.fastq.gz
INPUT_Z4ChIP_Rep12hr_R2.fastq.gz
INPUT_Z4ChIP_Rep20hr_R1.fastq.gz
INPUT_Z4ChIP_Rep20hr_R2.fastq.gz
INPUT_Z4ChIP_Rep22hr_R1.fastq.gz
INPUT_Z4ChIP_Rep22hr_R2.fastq.gz
INPUT_Z4ChIP_Rep30hr_R1.fastq.gz
INPUT_Z4ChIP_Rep30hr_R2.fastq.gz
INPUT_Z4ChIP_Rep32hr_R1.fastq.gz
INPUT_Z4ChIP_Rep32hr_R2.fastq.gz
SET1AB_ZC3H4ChIP_0hr.bw
SET1AB_ZC3H4ChIP_2hr.bw
NTDChIP_Rep10hr_R1.fastq.gz
NTDChIP_Rep10hr_R2.fastq.gz
NTDChIP_Rep12hr_R1.fastq.gz
NTDChIP_Rep12hr_R2.fastq.gz
NTDChIP_Rep20hr_R1.fastq.gz
NTDChIP_Rep20hr_R2.fastq.gz
NTDChIP_Rep22hr_R1.fastq.gz
NTDChIP_Rep22hr_R2.fastq.gz
NTDChIP_Rep30hr_R1.fastq.gz
NTDChIP_Rep30hr_R2.fastq.gz
NTDChIP_Rep32hr_R1.fastq.gz
NTDChIP_Rep32hr_R2.fastq.gz
INPUT_NTDChIP_Rep10hr_R1.fastq.gz
INPUT_NTDChIP_Rep10hr_R2.fastq.gz
INPUT_NTDChIP_Rep12hr_R1.fastq.gz
INPUT_NTDChIP_Rep12hr_R2.fastq.gz
INPUT_NTDChIP_Rep20hr_R1.fastq.gz
INPUT_NTDChIP_Rep20hr_R2.fastq.gz
INPUT_NTDChIP_Rep22hr_R1.fastq.gz
INPUT_NTDChIP_Rep22hr_R2.fastq.gz
INPUT_NTDChIP_Rep30hr_R1.fastq.gz
INPUT_NTDChIP_Rep30hr_R2.fastq.gz
INPUT_NTDChIP_Rep32hr_R1.fastq.gz
INPUT_NTDChIP_Rep32hr_R2.fastq.gz
SET1ABRNAPIINTD0hr.bw
SET1ABRNAPIINTD2hr.bw
H3K4ChIP_Rep10hr_R1.fastq.gz
H3K4ChIP_Rep10hr_R2.fastq.gz
H3K4ChIP_Rep12hr_R1.fastq.gz
H3K4ChIP_Rep12hr_R2.fastq.gz
H3K4ChIP_Rep14hr_R1.fastq.gz
H3K4ChIP_Rep14hr_R2.fastq.gz
H3K4ChIP_Rep124hr_R1.fastq.gz
H3K4ChIP_Rep124hr_R2.fastq.gz
H3K4ChIP_Rep20hr_R1.fastq.gz
H3K4ChIP_Rep20hr_R2.fastq.gz
H3K4ChIP_Rep22hr_R1.fastq.gz
H3K4ChIP_Rep22hr_R2.fastq.gz
H3K4ChIP_Rep24hr_R1.fastq.gz
H3K4ChIP_Rep24hr_R2.fastq.gz
H3K4ChIP_Rep224hr_R1.fastq.gz
H3K4ChIP_Rep224hr_R2.fastq.gz
H3K4ChIP_Rep30hr_R1.fastq.gz
H3K4ChIP_Rep30hr_R2.fastq.gz
H3K4ChIP_Rep32hr_R1.fastq.gz

	H3K4ChIP_Rep32hr_R2.fastq.gz<br>H3K4ChIP_Rep34hr_R1.fastq.gz<br>H3K4ChIP_Rep34hr_R2.fastq.gz<br>H3K4ChIP_Rep324hr_R1.fastq.gz<br>H3K4ChIP_Rep324hr_R2.fastq.gz<br>Input_H3K4ChIP_Rep10hr_R1.fastq.gz<br>Input_H3K4ChIP_Rep10hr_R2.fastq.gz<br>Input_H3K4ChIP_Rep12hr_R1.fastq.gz<br>Input_H3K4ChIP_Rep12hr_R2.fastq.gz<br>Input_H3K4ChIP_Rep14hr_R1.fastq.gz<br>Input_H3K4ChIP_Rep14hr_R2.fastq.gz<br>Input_H3K4ChIP_Rep124hr_R1.fastq.gz<br>Input_H3K4ChIP_Rep124hr_R2.fastq.gz<br>Input_H3K4ChIP_Rep20hr_R1.fastq.gz<br>Input_H3K4ChIP_Rep20hr_R2.fastq.gz<br>Input_H3K4ChIP_Rep22hr_R1.fastq.gz<br>Input_H3K4ChIP_Rep22hr_R2.fastq.gz<br>Input_H3K4ChIP_Rep24hr_R1.fastq.gz<br>Input_H3K4ChIP_Rep24hr_R2.fastq.gz<br>Input_H3K4ChIP_Rep224hr_R1.fastq.gz<br>Input_H3K4ChIP_Rep224hr_R2.fastq.gz<br>Input_H3K4ChIP_Rep30hr_R1.fastq.gz<br>Input_H3K4ChIP_Rep30hr_R2.fastq.gz<br>Input_H3K4ChIP_Rep32hr_R1.fastq.gz<br>Input_H3K4ChIP_Rep32hr_R2.fastq.gz<br>Input_H3K4ChIP_Rep34hr_R1.fastq.gz<br>Input_H3K4ChIP_Rep34hr_R2.fastq.gz<br>Input_H3K4ChIP_Rep324hr_R1.fastq.gz<br>Input_H3K4ChIP_Rep324hr_R2.fastq.gz<br>SET1ABH3K4me30hrdownsampledMERGE.bw<br>SET1ABH3K4me32hrdownsampledMERGE.bw<br>SET1ABH3K4me34hrdownsampledMERGE.bw<br>SET1ABH3K4me324hrdownsampledMERGE.bw
Genome browser session (e.g. UCSC)	No link available

Methodology

Replicates	All ChIP-seq experiments were performed in biological triplicates
Sequencing depth	All libraries were sequenced as 40bp paired-end reads. Number of reads is given in Supplementary Table.
Antibodies	anti-H3K4me3 Klose Lab N/A<br>anti-T7 Cell Signalling D9E1X<br>anti-Rbp1-NTD Cell Signalling D8L4Y
Peak calling parameters	H3K4me3 peaks were called using MACS2 ('BAMPE' and 'broad' options specified) with corresponding input samples for background normalization.
Data quality	Quality of ChIP-seq data was assessed by visual inspection of individual replicate bigWig files and comparison with other published data sets, as well as by metaplot, heatmap and correlation analysis using deepTools.
Software	Paired-end reads were aligned to the concatenated mouse (mm10) and spike-in (dm6 for native, hg19 for cross-linked cChIP-seq) genome sequences using Bowtie 2 ("–no-mixed" and "–no-discordant" options). Only uniquely mapped reads were kept for downstream analysis, after removal of PCR duplicates with Sambamba. Genome coverage tracks were generated using the pileup function from MACS2. Metaplot and heatmap analysis of ChIP-seq read density at regions of interest was performed with computeMatrix and plotProfile/plotHeatmap from deepTools.
