# Supplementary material for: A CpG island-encoded mechanism protects genes from premature transcription termination
Source: Nat Commun. 2023 Feb 9;14:726. doi: 10.1038/s41467-023-36236-2 (PMC9911701; doi:10.1038/s41467-023-36236-2)
Supplement: Supplementary file 6 — Source Data [file 41467_2023_36236_MOESM6_ESM.zip › Source Data - Western Blots.pdf]

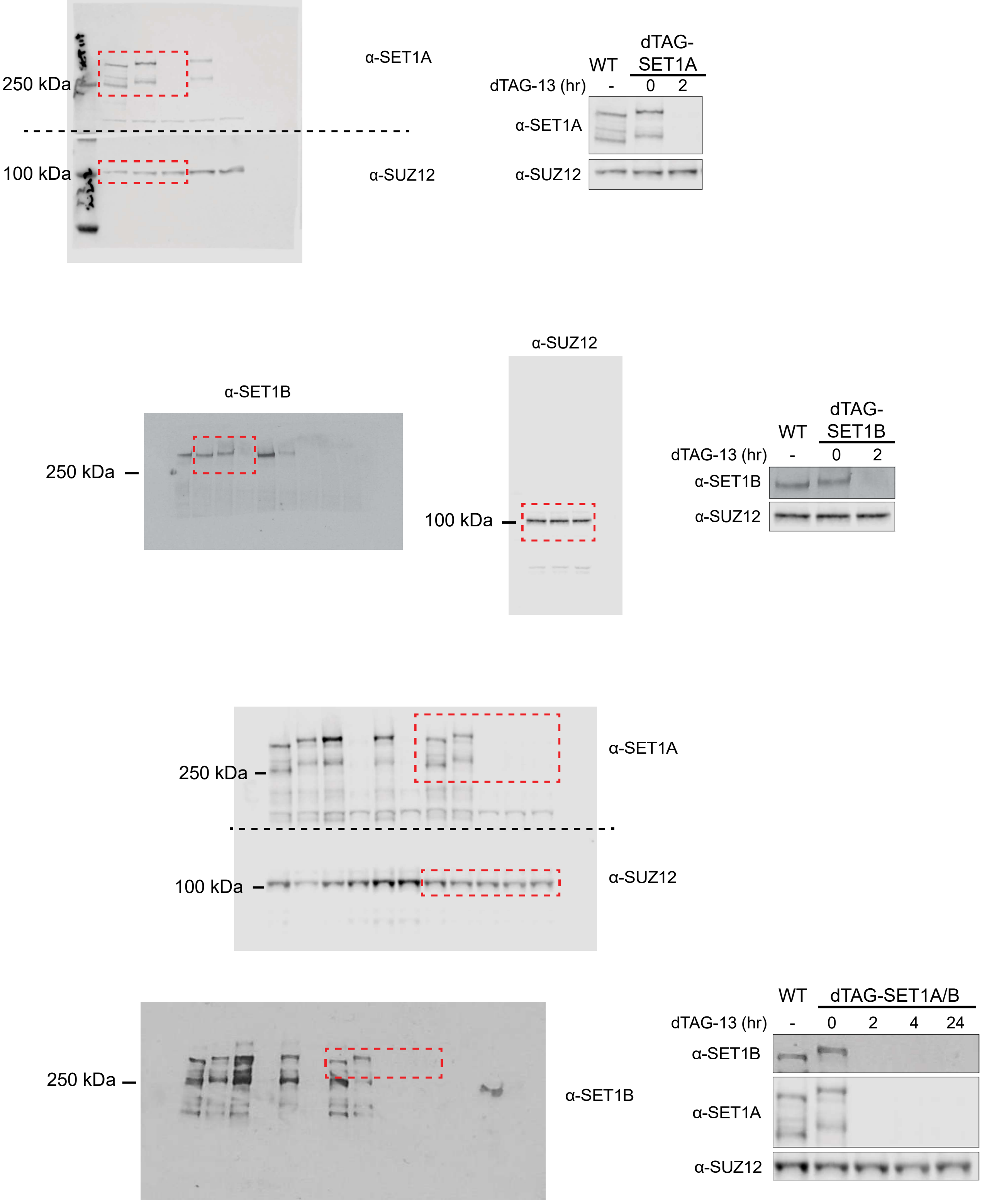
250 kDa
100 kDa
α-SET1A
α-SUZ12
WT
dTAG-SET1A
dTAG-13 (hr)
-
0
2
α-SET1A
α-SUZ12
α-SET1B
250 kDa
α-SUZ12
100 kDa
WT
dTAG-SET1B
dTAG-13 (hr)
-
0
2
α-SET1B
α-SUZ12
250 kDa
α-SET1A
100 kDa
α-SUZ12
250 kDa
α-SET1B
WT
dTAG-SET1A/B
dTAG-13 (hr)
-
0
2
4
24
α-SET1B
α-SET1A
α-SUZ12

Figure 1

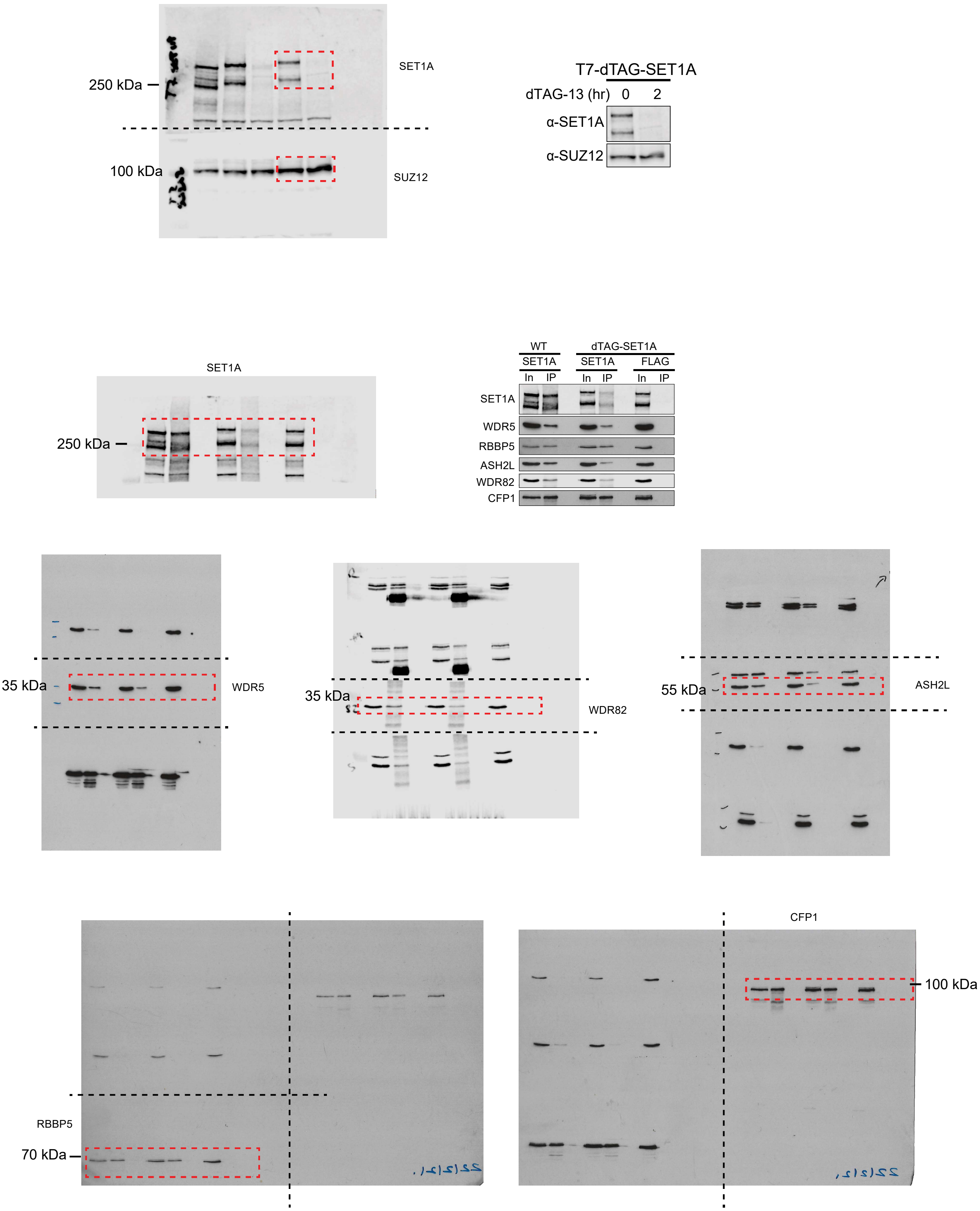

Supplementary Figure 1

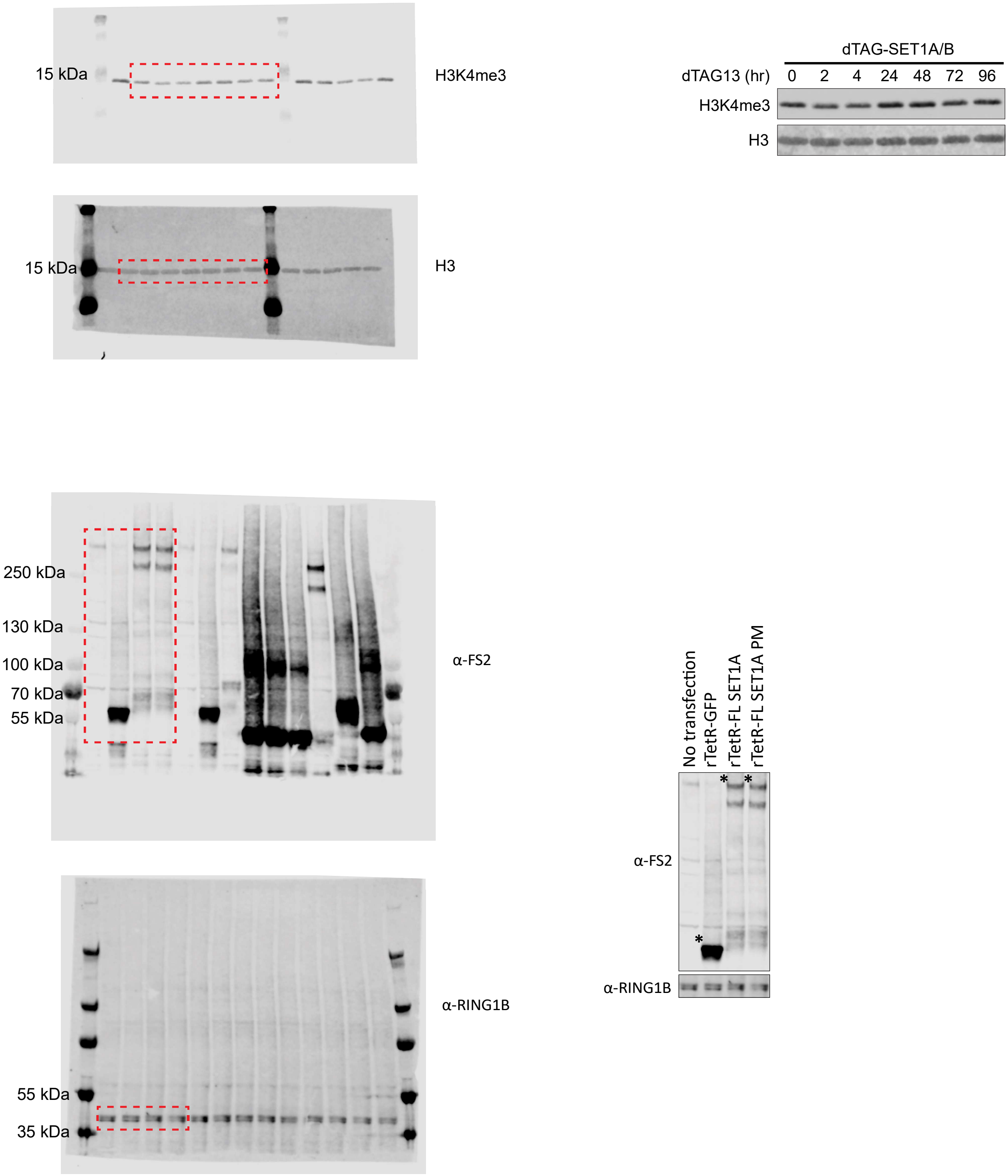

Supplementary Figure 2

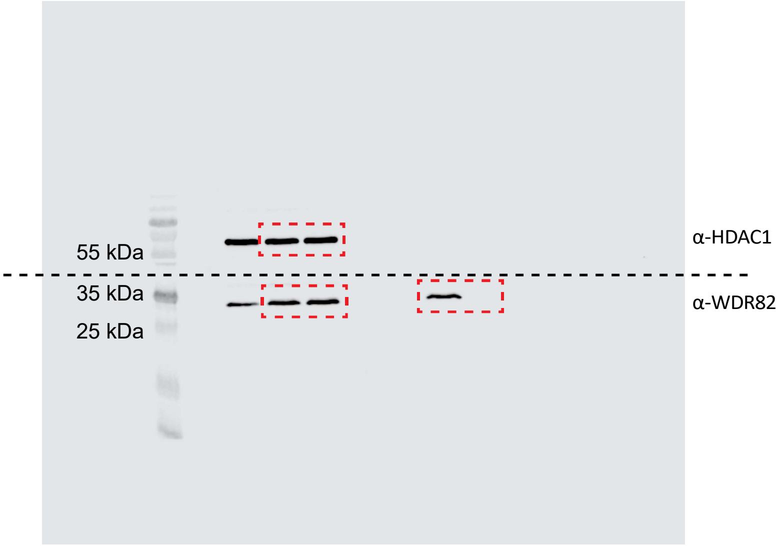

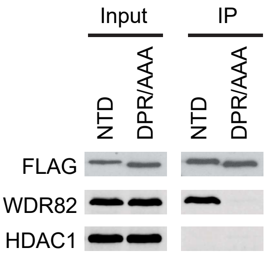

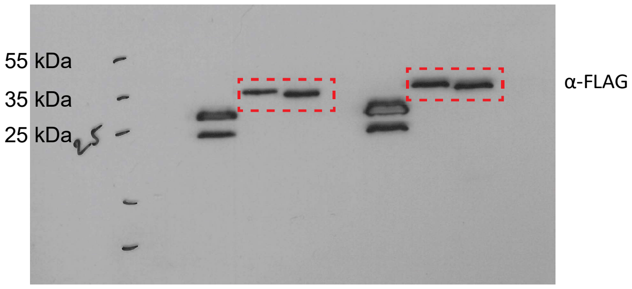

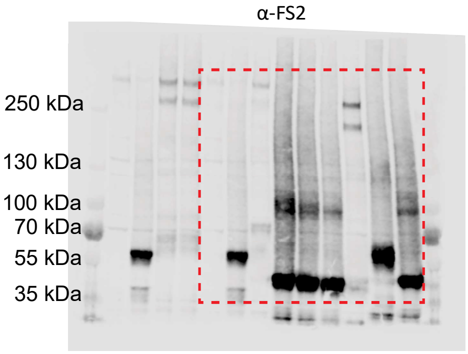

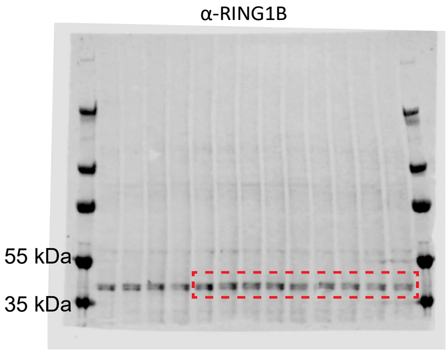

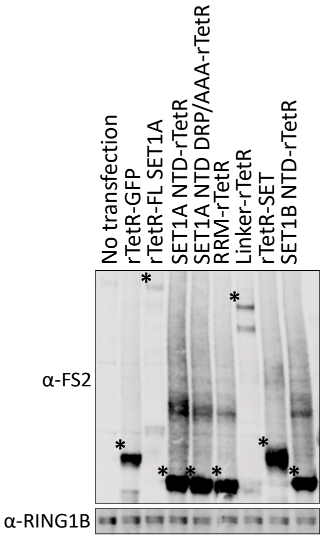

Figure 3 and Supplementary Figure 3

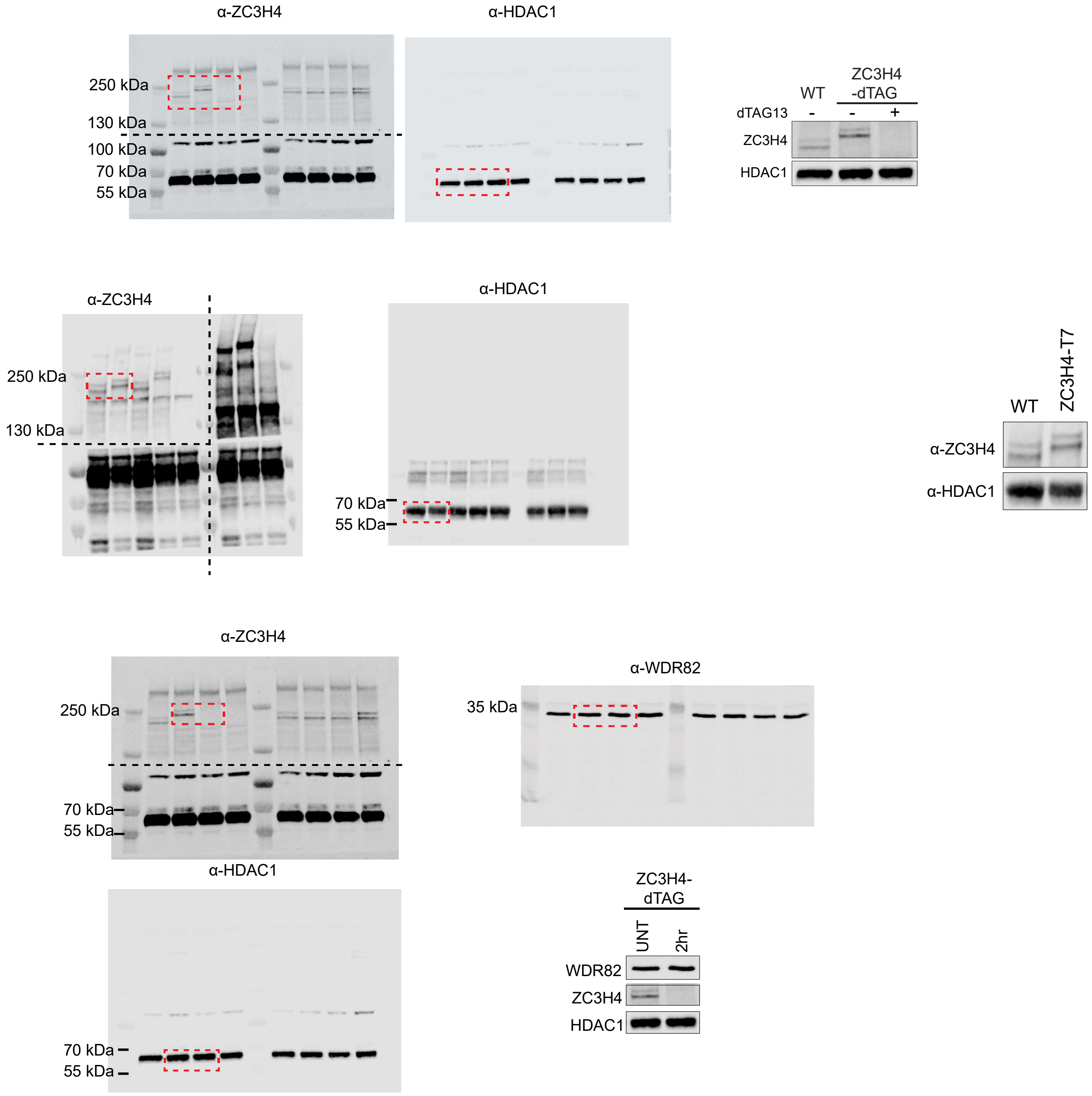

Figure 5 and Supplementary Figure 5

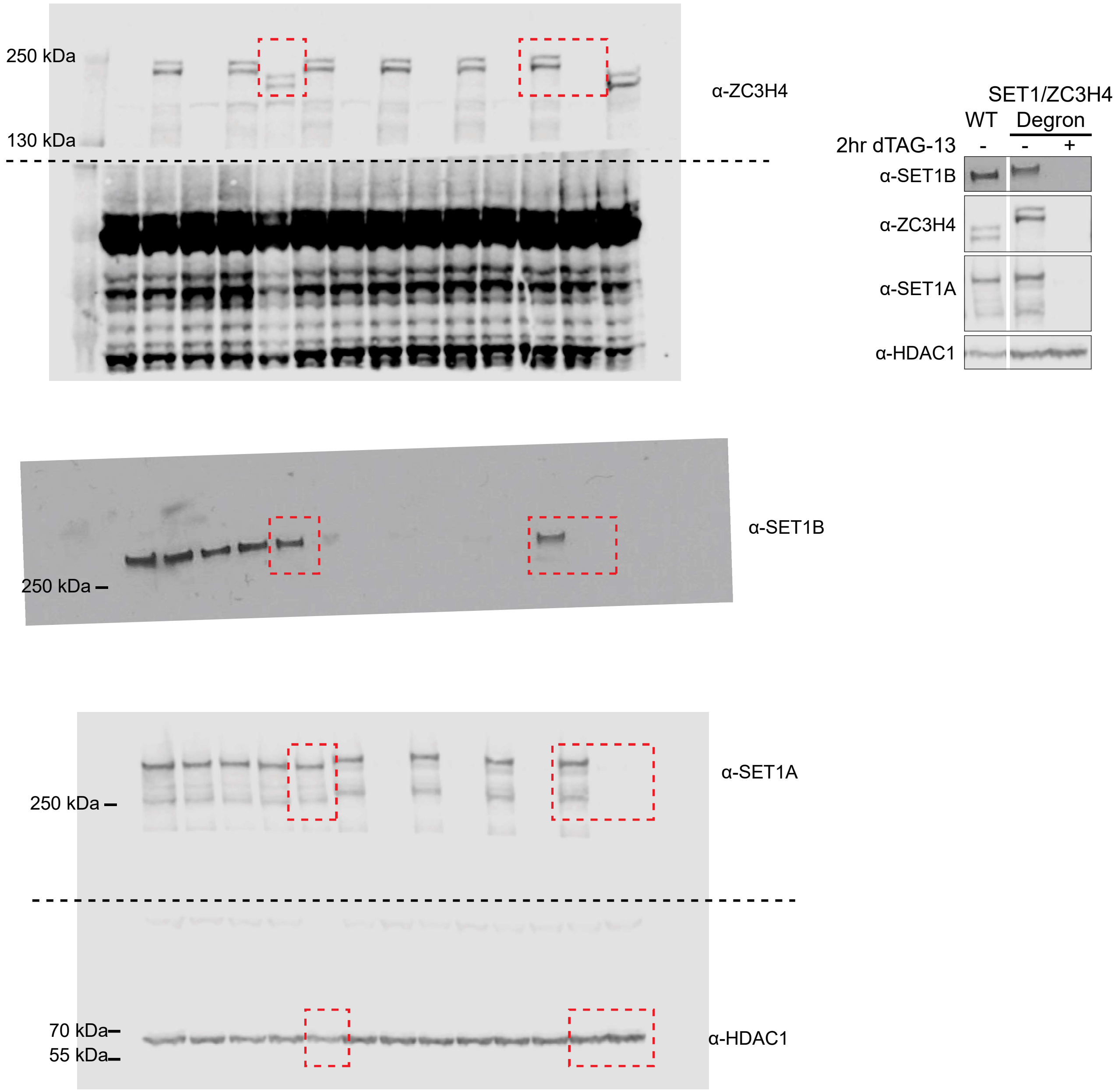

Supplementary Figure 6C

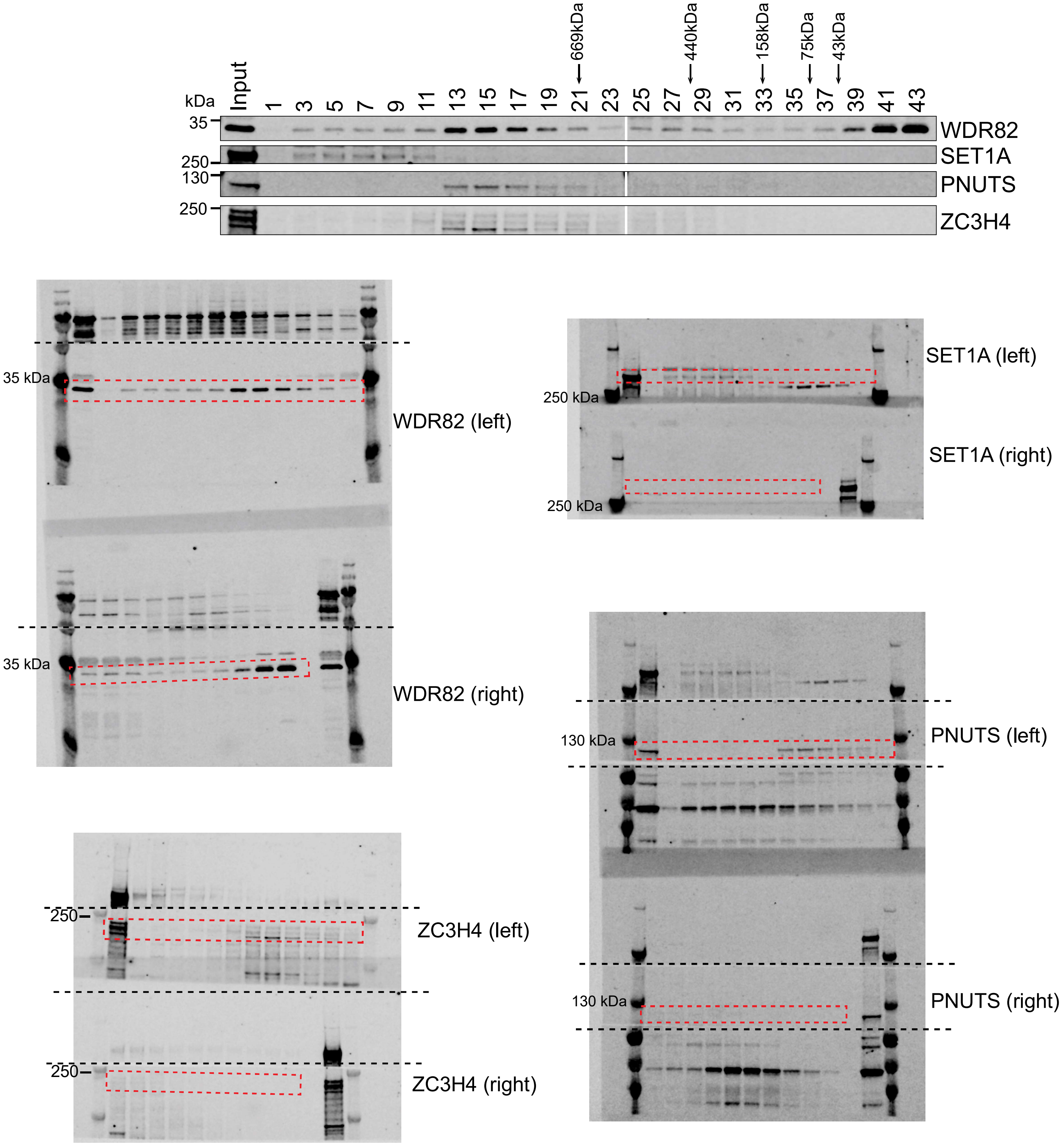

Supplementary Figure 6H
